# The Effect of Cosmetic Surgery on Sexual Self-Esteem: Attitudes toward Body Image and Well-Being in Married Women

**DOI:** 10.29252/wjps.9.2.153

**Published:** 2020-05

**Authors:** Ladan Esmalian Khamseh, Mahmood Nodargahfard

**Affiliations:** 1 Faculty of Psychology, Islamic Azad University, North Tehran Branch, Tehran, Iran;; 2 Faculty of Psychology, Islamic Azad University, Karaj Branch, Karaj, Iran

**Keywords:** Sexual self-esteem, Body image, Well-being, Cosmetic surgery

## Abstract

**BACKGROUND:**

Nowadays in different communities, we are confronting an ever-increasing trend of cosmetic surgeries. The present research was carried out with the aim of investigating sexual self-esteem, attitude to body image and well-being in married women aged from 20 to 30 years old before and after cosmetic surgery in Tehran, Iran.

**METHODS:**

The research was a semi-experimental design with pre- and post-test. The statistical population consisted 80 married women. The research sample was selected by means of the convenience sampling approach. This group was similar in terms of age, education, and marital status. The research data were accumulated using the Zeanah and Schwarz sexual self-esteem questionnaire, the Fisher body image questionnaire and the Ryff psychological well-being questionnaire, and were analyzed by multivariate analysis of variance.

**RESULTS:**

The average scores of all three variables, involving “sexual self-esteem”, “body image” and “well-being” were dramatically different before and after cosmetic surgery (*p*<0.05).

**CONCLUSION:**

It can be ascertained that people after cosmetic surgery had higher sexual self-esteem, were more satisfied with their body image, and experienced more well-being.

## INTRODUCTION

The aspiration for beauty is a category that is as longstanding as the creation of humanity. Human beings have constantly sought beauty and have been looking for fixing their defects; furthermore, this desire for beauty and grace has always provoked humans to undergo cosmetic surgeries, so that the first cosmetic surgeries have been reported in Egypt. Hence, we can admit that cosmetic surgery is not an emerging phenomenon.^1^ Also, in the present age, cosmetic surgeries have achieved a large proportion of surgeries, with the outcome that, the British Association of Aesthetic Plastic Surgeons have announced 34 percent increase in cosmetic surgeries.^2^

Besides, in Norway, 7 percent of women have been subjected to at least one type of cosmetic surgery.^3^ This phenomenon is not just restricted to European countries. American Board of Cosmetic Surgery also released a statistic indicating that cosmetic surgery has increased by 77 percent from 2000 to 2012.^4^ The rate of cosmetic surgery in Iran has progressively increased, as well.^5^ Moreover, the observations suggest that only 10 percent of the surgeries have been performed on account of impaired function and abnormal appearance, and other cases are just accomplished for the sake of more outer beauty.^6^

Along with this rise, finding the motivation and the reason behind the decision to have cosmetic surgery are of great importance. Indeed, psychological components have an influential role in the demand for cosmetic surgery, as well as in the prognosis of surgery and postoperative satisfaction.^7^ The existence of problems, such as body dysmorphic disorder, low self-esteem and even symptoms of psychosis can lead a person to undergo surgery; consequently, after completing the evaluation of physical examinations. The individual’s psychological health assessment for surgery is the most prominent action before holding the surgical knife.^8^

In this respect, the current study explored the psychological features incorporating sexual self-esteem, body image, and well-being among people with a history of cosmetic surgery and normal individuals. One of the components that can be examined in this regard is the body image. The body image can be delineated as an individual perspective and the result of the experience of one’s own body,^9^ and for the first time in 1920, Shilder has characterized it as follows with a psychological perspective: “an image of our body that we create in our minds, and the way in which the body revealed itself to us”.^10^

The body image can be influenced by factors such as social media, family and friends, accidents, physical development, etc. In addition, any weaknesses can adversely affect the body image.^11^ The negative image is associated with poor self-esteem,^12^ negative emotions^13^and body dysmorphic disorder.^14^ A group of research studies in the field of the assessment of the relationship between negative body image demonstrated that women with negative images toward their bodies are more inclined to change their appearance through cosmetic surgery.^15^

However, another study implies that those undergoing cosmetic surgeries typically do not increase satisfaction for body image; therefore, the relationship between the cosmetic surgery and body image has always been controversial.^16^ But another variable that can be effective is sexual self-esteem that is defined as a person’s overall desire for a positive evaluation of his capacity to engage in healthy sexual behaviors and sexual experiences healthily and satisfactorily.^17^ Sexual self-esteem contains five elements encompassing skill and experience, attractiveness, control, moral judgment, and adaptation.^18^

When sexual self-esteem is hurt, the individual’s opinion of oneself, life satisfaction, pleasure experience and interaction with others encounter damage, and in case of severe ruin, impairment on one’s performance is caused.^19^ The most important requirement for good self-esteem in people is the positive perception of physical appearance they have in the opinion of their family and friends.^20^ People with low sexual self-esteem have problems with sexual and marital issues and also have higher rates of performance deficiency.^21^

Sexual self-esteem and body image are both considered as the important psychological factors in the sexual function.^22^ Nowadays, a new perspective is emerging in the health-related sciences. This scientific insight concentrates on the positive aspects of health and psychological well-being.^23^ Psychological well-being denotes to the emotional and cognitive evaluation of one’s life. Psychological well-being involves experiencing pleasant emotions, low levels of negative mood and high life satisfaction.^24^

From the point of view of scientists, an essential part of life is an intrinsic feeling of psychological well-being through which everyone loves his life.^25^ The feeling of being ugly and receiving cosmetic surgery are mostly affected by people’s minds, and beliefs play a major role in its creation, so that, people with irrational beliefs like inferiority feeling that usually have less psychological well-being.^26^ Components of psychological well-being include self-acceptance, positive relationships with others, independency, environmental mastery, purposeful life and personal growth.^27^

Considering the importance and necessity of further studies in order to understand the psychological factors influencing the propensity to undergo cosmetic surgery and with respect to the high rates of cosmetic surgery in our community, the present study was an attempt at understanding and recognizing more things about the psychological differences between people with a history of cosmetic surgery and normal people.

## MATERIALS AND METHODS

The present study used a quasi-experimental design with a pre- and post-test. The study was approved in the institution ethics committee. The statistical population of the study comprised 80 married women between 20 and 30 years old. The group was chosen by convenience sampling and were matched by terms of age, education and marital status. Definitely, all subjects met the inclusion criteria including age, marital status, not undergoing cosmetic surgery for pre-test, and 4-5 months after surgery to reduce swelling for post-test. A written consent was provided from each patient.

By referring to the Mehran Beauty Clinic in Tehran, Iran and making a list of people who were candidate for cosmetic surgery, research information was gathered and its implementation method was fulfilled. Before initiating the research, ethical considerations were described for the individuals in an exclusive session, and the nature and mode of cooperation in conducting the research were introduced to them. Moreover, they were allowed to opt out of continuing cooperation at each stage of the study. In this research work, all patient’s information was kept confidential and was only accessible to the researcher. 

The following instruments were used to collect data in complement to the demographic questionnaire including (i) Sexual Self-Esteem Questionnaire (SSEI-W-SF) that contained 35 items for inspecting women’s sexual self-esteem. Questions were answered on a 6-point Likert scale ranging from 1 (completely disagree) to 6 (completely agree). Questions (6-7-11-13-14-17-18-20-21-22-23-26-27-28-29-31-34) were scored in the reverse order.^28^

This Questionnaire comprised dimensions incorporating skill and experience, attractiveness, control, moral judgment, and adaptation. Cronbach’s alpha coefficients of its subscales were found to be 0.84 for skill and experience, 0.88 for attractiveness, 0.80 for control, 0.80 for moral judgment, 80% for adaptation and 0.92 for the total questionnaire.^28^ The validity of the sexual self-esteem questionnaire was considered to be 0.57.^28^ In Iran, the reliability coefficient was assessed to be 0.91 and the validity to be 0.54 for the whole scale in 2014.^21^

(ii) The questionnaire developed by Fisher for each item had a value ranging from 1 to 5 (very dissatisfied=1, dissatisfied=2, moderate=3, satisfied=4, very satisfied=5). This test had 46 items, where earning a score of 46 on this test denoted to impairment, and a rating higher than 46 (up to 230) indicated no impairment. The validity and reliability of this test was considered 86%. On the other hand, the Cronbach’s alpha coefficient was 93/60 to be optimal for content validity. 

(iii) Carole Ryff’s Psychosocial Well-being Questionnaire measured psychological well-being scales. The initial version of the questionnaire consisted of 84 items, and then 54-item version and finally the short 18-item form were designed. In the meantime, this research employed the 18-item form. A 6-point scoring method (ranging from -1=completely disagree to +6=completely agree) was specified for it. Reverse grading was utilized for questions as reported before (3-4-5-9-10-13-16-17).^29^

The reliability of this questionnaire was reported to be numerous, with the result that their Cronbach’s alpha was between 0.77 and 0.90.^29^ This test was normalized on students of Tehran University for the first time and reported the internal consistency of reliability to be 0.94 for the whole test.^30^ In another research, the validity of the questionnaire was shown to be from 0.89 to 0.90 and obtained the value of Cronbach’s alpha of the questionnaire as 0.69.^31^

## RESULTS

The present research investigated 80 studied subjects, before and after cosmetic surgery. Table 1 reveals the mean and standard deviation for scores of different scales and sub-scales before and after cosmetic surgery. Results regarding the different dimensions of sexual self-esteem clarified that for all the aspects of sexual self-esteem including experience and skill, attractiveness, control, adaptation, and moral judgment, the mean score of people after surgy was higher than before surgery. Results pertaining to the two variables of facial components and the body image, the total score confirmed that the mean score after surgery was higher than before (Tables 1-4). Results related to the variables of well being such as independency, environmental mastery, personal growth, positive relationship, self-acceptance, life purposefulness, and the total score of well-being disclosed that in all of these cases, the total score of people after surgery was higher than before (Table 1).

**Table 1:** Mean and standard deviation of before and after surgy experienced by populations in terms of different scales and sub-scales of the research

**Dependent variable**	**Before surgery**		**After surgery **	
	**Mean**	**Standard deviation**	**Mean**	**Standard deviation**
Experience and skills	15.08	2.40	19.20	2.56
Attractiveness	16.30	2.17	19.30	2.23
Control	15.98	3.00	19.05	2.60
Adaptation	17.25	2.79	19.40	2.76
Moral judgment	15.75	2.32	18.58	1.92
Total score of sexual self esteem	80.35	6.99	95.53	5.67
Facial components	26.80	4.69	32.25	5.23
Total score of body image	170.13	19.32	180.58	24.13
Independency	13.43	2.35	15.40	3.33
Environmental mastery	14.08	2.22	14.48	3.01
Personal growth	12.48	2.11	14.20	2.93
Positive relationship	10.18	3.06	12.78	3.34
Self acceptance	12.45	1.93	14.08	2.34
Life purposefulness	10.38	2.44	13.98	2.88
Total score of well-being	72.98	8.47	48.90	13.71

**Table 2:** Multivariate analysis of variance for scores of measurement variables before and after surgery

**Index**	**Value**	**F**	**Df hyposesis**	**Df error**	**P**	**Eta squared**
Pillai’s Trace	0.63	42.86	3	76	0.001	0.629
Wilks Lambda	0.37	42.86	3	76	0.001	0.629
T Hotelling	1.69	42.86	3	76	0.001	0.629
Biggest square root	1.69	42.86	3	76	0.001	0.629

**Table 3:** Analysis of variance in order to measure the effects between the two groups for the measured variables

**Dependent variable**	**Total squares**	**Df**	**Mean squares**	**F**	**P**	**Eta coefficient**
Sexual self-esteem	4605.61	1	4605.61	113.79	0.000	0.593
Body image	2184.05	1	2184.05	4.57	0.036	0.055
Well being	2844.11	1	2844.11	21.91	0.000	0.219

**Table 4:** Comparing the means of the two experimental and control groups for measured traits

**Dependent variable**	**Group**	**Standard error± mean**	**Average difference**	**P**
Self-esteem	After surgery Before surgery	95.53±1.01 80.35±1.01	15.17	0.001
Body Iimage	After surgery Before surgery	180.58±3.46 170.13±3.46	10.45	0.063
Well being	After surgery Before surgery	84.9±1.8 72.98±1.8	11.93	0.001

## DISCUSSION

The results confirmed that there was a significant difference between before and after surgery regrading the sexual self-esteem, so that women after cosmetic surgery reported higher sexual self-esteem. These findings are in line with surveys performed before^32^^-^^34^ The results of the present study illustrated that cosmetic surgery provided a positive effect on people’s self-esteem and in particular on their sexual self-esteem. In spite of the fact that sexual self-esteem can rise in adulthood, there is also the potential to be lost.^35^

The major part of what happens to self-esteem is attributed to how one faces the challenges of life. People who are seeking cosmetic surgery, frequently have a negative self-image, and in fact, the negative image is an important factor for undergoing cosmetic surgery. In addition, since there is a strong correlation between self-esteem and the negative image, perhaps one of these challenges is to have such a negative self-image, which endangers sexual self-esteem. However, people usually feel good about their sexual self-esteem after being subjected to cosmetic surgery; in order that, former research works demonstrated positive changes in people’s sexual self-esteem after being subjected to cosmetic surgery that are still stable even after 6 months of follow-up.^35^

Another finding of the present study was a reducing effect of cosmetic surgery regarding the score of the negative self-image in in people after cosmetic surgery, which is in accordance with researches carried out before.^36^^-^^38^ One of the issues that always comes up about body image is person’s self-evaluation. Findings from this study suggested promoting a positive attitude towards body and self-image after getting cosmetic surgery. One of the explanations that can be made for this issue is that the person receives positive feedback from others in his social interactions; consequently, gradually concludes that his positive change after surgery has caused him to enjoy a great deal of privileges in the community and to realize the importance of cosmetic surgery regarding this achievement more than ever.

However, among the other examined components in this research, well-being can be cited, that according to the achieved results, a noticeable difference was observed after and before cosmetic surgery. The results of this research are in agreement with other researches.^39^^-^^41^ People who visit the clinic for cosmetic surgery often suffer from stress and have anxiety symptoms and even depression symptoms; furthermore, they recurrently have low body image and low self-esteem. People, who have not undergone any cosmetic surgery, have higher levels of frustration and negative self-evaluation.^39^^-^^41^

Given that in today’s society, a huge emphasis is placed on attractiveness and physical fitness, and this matter has a profound effect on people’s self-evaluation and brings on a substantial decrease in people’s well-being. Whereas, people with a history of cosmetic surgery report lower anxiety and depression, and of course, enjoy higher well-being. In accordance with the achieved results, it can be claimed that despite all the dangers that cosmetic surgery can cause, it also has positive effects. In compliance with the data obtained from the study, cosmetic surgery can be effective in enhancing multiple psychological factors such as the body image, and on the other hand, the remarkable role of cosmetic surgery in improving physical anomalies should not be ignored.
